# Comparative Ochratoxin Toxicity: A Review of the Available Data

**DOI:** 10.3390/toxins7104253

**Published:** 2015-10-22

**Authors:** Alexandra H. Heussner, Lewis E. H. Bingle

**Affiliations:** 1 Human and Environmental Toxicology, University of Konstanz, Universitätsstrasse 10, 78457 Konstanz, Germany; 2 Department of Pharmacy, Health and Well-Being, University of Sunderland, City Campus, Sunderland SR1 3SD, UK; E-Mail: lewis.bingle@sunderland.ac.uk

**Keywords:** ochratoxin, biosynthesis, metabolites, detection, comparative toxicity

## Abstract

Ochratoxins are a group of mycotoxins produced by a variety of moulds. Ochratoxin A (OTA), the most prominent member of this toxin family, was first described by van der Merwe *et al.* in Nature in 1965. Dietary exposure to OTA represents a serious health issue and has been associated with several human and animal diseases including poultry ochratoxicosis, porcine nephropathy, human endemic nephropathies and urinary tract tumours in humans. More than 30 years ago, OTA was shown to be carcinogenic in rodents and since then extensive research has been performed in order to investigate its mode of action, however, this is still under debate. OTA is regarded as the most toxic family member, however, other ochratoxins or their metabolites and, in particular, ochratoxin mixtures or combinations with other mycotoxins may represent serious threats to human and animal health. This review summarises and evaluates current knowledge about the differential and comparative toxicity of the ochratoxin group.

## 1. Ochratoxin A—An Important Mycotoxin

Ochratoxin A was first described in a paper published in Nature by van der Merwe and co-workers (1965) after they isolated a new toxic metabolite from *Aspergillus ochraceus* [1]. Ochratoxins are produced by certain *Aspergillus* species such as *A. ochraceus* or *A. niger* and some *Penicillium* species, especially *P. verrucosum*. The main forms are ochratoxin A, B, and C, which differ in that ochratoxin B (OTB) is a non-chlorinated form of ochratoxin A (OTA) and ochratoxin C (OTC) is an ethyl ester of OTA (Table 1). OTA ((R)-*N*-((5-Chlor-3,4-dihydro-8-hydroxy-3-methyl-1-oxo-1*H*-benzo[c]-pyran-7-yl)-carbonyl)-3-phenylalanin) is the most prevalent and relevant form of this group, while OTB and OTC are generally assumed to be of lesser importance [2].

**Table 1:** Overview of main ochratoxin forms.

Name	OTA	OTB	OTC
CAS number	303-47-9	4825-86-9	4865-85-4
Molecular formula	C_20_H_18_ClNO_6_	C_20_H_19_NO_6_	C_22_H_22_ClNO_6_
Molar mass (g·mol^−1^)	403.8	369.4	431.9

CAS, Chemical Abstracts Service.

OTA is a highly abundant food and animal feed contaminant and is frequently detected in all types of cereals and cereal products, but also coffee, cacao, grapes, raisins, wine, soy, spices, nuts, pulses, liquorice and beer [3,4,5,6]. A European report estimated the contributions to the total human adult OTA exposure as being 44% cereals, 15% others, 10% wine, 9% coffee, 7% beer, 5% cacao, 4% dried fruits, 3% meat and 3% spices [5]. In general, the mean OTA contamination levels in European food commodities are relatively low (ng kg^−1^ to µg kg^−1^), however, elevated concentrations can occur in individual batches. Even though preventive measures are taken to keep the levels of OTA in food low, a certain degree of contamination seems unavoidable [7,8]. Furthermore, in other countries where screening of food is rare and outdated storage and transport conditions are still in use, much higher contamination levels may occur. Many farm animals are fed with cereal-based diets exposing them to ochratoxins (usually in the low to high µg kg^−1^ range) [9], but again elevated contamination levels may occur depending on storage and feeding practice [10]. The highest contamination level reported was 80 mg OTA kg^−1^ in mouldy bread intended for animal feeding [11]. OTA has a high affinity for proteins, particularly serum albumin, which promotes bio-accumulation in the organs of animals, leading to carry-over of the contamination [9]. Therefore, muscle and offal products, as well as milk and sometimes eggs, may be contaminated, and the highest level of OTA contamination can be found in porcine blood-based sausages, “black pudding”, and liver products (e.g., pâtés, sausages) [9].

OTA-related diseases of economic importance occur in poultry (poultry ochratoxicosis) [12,13,14,15,16,17] and pigs (mycotoxic porcine nephropathy, MPN) [18,19,20,21]. These diseases are characterised by severe kidney damage, which could be clearly associated with the exposure to ochratoxins, sometimes in combination with other mycotoxins, as shown in several epizootiological and feeding studies [20,21,22,23,24,25]. Similarly, a slow, progressive renal disease (Endemic Nephropathy, EN) occurs in humans which is characterised by cellular interstitial fibrosis, tubular atrophy, and karyomegaly predominately in proximal convoluted tubules [26,27,28,29,30]. The aetiology of EN is still unknown, but based on epidemiological features, researchers agree that the causative agent is of natural origin [26,31]. There are several hypotheses as to the causes of EN that are currently in circulation, the two most important focus on the potential roles of aristolochic acid from the plant birthwort (*Aristolochia clematitis*) and mycotoxins (OTA and citrinin).

The toxicity of OTA has been investigated intensively in recent decades and numerous comprehensive reviews have been published with respect to all types of toxic effects. The scope of this review is to summarise and evaluate the existing data on the less well-studied members of the ochratoxin family, including the known metabolites, in comparison to OTA.

## 2. Occurrence of Ochratoxins

### 2.1. Biosynthesis

The biosynthetic pathway of OTA (an amide formed between phenylalanine and a chlorinated dihydroisocoumarin) is not completely established. However, it is known that the phenylalanine moiety originates from the shikimate pathway and the dihydroisocoumarin moiety from a polyketide (pentaketide) pathway, with the latter differing between *Penicillium* and *Aspergillus* species [32,33,34,35,36,37,38,39]. Also, the OTA polyketide synthase genes have been characterised from several fungal species [32,40,41,42,43].

Both ochratoxin production and the primary producing organisms depend on factors that affect fungal physiology such as temperature, moisture and the available substrates [2,20,33,39,44]. *Aspergillus* species can produce OTA and OTB in parallel [44,45], and experiments with *A. ochraceus* have revealed growth-associated production of OTA and OTB, in which the yield and the ratio were dependent on the prevailing culture conditions [44,46,47,48]. Often, the amount of OTB produced was considerably lower than that of OTA, but under certain conditions the level of OTB production was comparable to that of OTA [46,47]. The reported production ratios (OTA:OTB) ranged from 2:1 to 34:1 [46,49,50]. A complex interaction of different carbon sources, basal media and nitrogen sources seems to be crucial [44]. An increased OTA production was correlated to an induction of OTA polyketide synthase expression, whereas OTB production does not correlate with transcription of the polyketide synthase gene [44]. Laboratory fermentation experiments with *A. ochraceus* resulted in very high yields (up to 10 mg/g) of OTA, OTB and transitorily also ochracin (mellein, see Table 2) [46]. The intermediate metabolite OTβ was found to be biotransformed very efficiently into both OTA and OTB (14% and 19%, respectively), whereas OTα was biotransformed only into OTA (4.9%). Also, OTB is poorly converted (1.5%) into OTA, whereas some OTB may be produced by dechlorination of OTA [46].

**Table 2:** Natural and synthetic forms of ochratoxins. 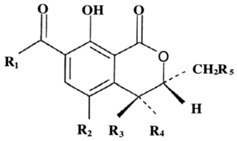

Name	Abbrev.	R1 and Others	R2	R3	R4	R5
***Naturally occurring ochratoxins*, *metabolites and conjugates***						
Ochratoxin A	OTA	Phenylalanyl	Cl	H	H	H
Ochratoxin B	OTB	Phenylalanyl	H	H	H	H
Ochratoxin C	OTC	Phenylalanyl, ethyl ester	Cl	H	H	H
Ochratoxin A methyl ester	--	Phenylalanyl, methyl ester	Cl	H	H	H
Ochratoxin B methyl ester	--	Phenylalanyl, methyl ester	H	H	H	H
Ochratoxin B ethyl ester	--	Phenylalanyl, ethyl ester	H	H	H	H
Ochratoxin α	OTα	OH	Cl	H	H	H
Ochratoxin β	OTβ	OH	H	H	H	H
5′-Hydroxyochratoxin A	5′-OH-OTA	Phenylalanyl, OH at C-5′	Cl	H	H	H
7′-Hydroxyochratoxin A	7′-OH-OTA	Phenylalanyl, OH at C-7′	Cl	H	H	H
9′-Hydroxyochratoxin A	9′-OH-OTA	Phenylalanyl, OH at C-9′	Cl	H	H	H
4*R*-Hydroxyochratoxin A	4*R*-OH-OTA	Phenylalanyl	Cl	H	OH	H
4*S*-Hydroxyochratoxin A	4*S*-OH-OTA	Phenylalanyl	Cl	OH	H	H
4*R*-Hydroxyochratoxin B	4*R*-OH-OTB	Phenylalanyl	H	H	OH	H
4*S*-Hydroxyochratoxin B	4*S*-OH-OTB	Phenylalanyl	H	OH	H	H
10-Hydroxyochratoxin A	10-OH-OTA	Phenylalanyl	Cl	H	H	OH
Open lactone of ochratoxin A	OP-OTA	Phenylalanyl	Cl	H	H	H
Ochratoxin hydroquinone	OTHQ	Phenylalanyl	OH	H	H	H
Ochratoxin quinone	OTQ	Phenylalanyl	O	H	H	H
Ochratoxin α glucuronide	--	Glucuronide	Cl	H	H	H
	--	OH; glucuronide at C-8	Cl	H	H	H
Ochratoxin A phenol-glucuronide	--	Phenylalanyl; glucuronide at C-8	Cl	H	H	H
Ochratoxin A amino-glucuronide	--	Phenylalanyl; glucuronide at N	Cl	H	H	H
Ochratoxin A acyl-glucuronide	--	Phenylalanyl; acyl-glucuronide instead of carboxyl	Cl	H	H	H
Ochratoxin A acyl-hexose	--	Phenylalanyl; acyl-hexose instead of carboxyl	Cl	H	H	H
Ochratoxin A acyl-pentose	--	Phenylalanyl; acyl-pentose instead of carboxyl	Cl	H	H	H
***Synthetic ochratoxins***						
Ochratoxin A, tyrosine analogue	--	Tyrosine	Cl	H	H	H
Ochratoxin A, serine analogue	--	Serine	Cl	H	H	H
Ochratoxin A, hydroxyproline analogue	--	Hydroxyproline	Cl	H	H	H
Ochratoxin A, lysine analogue	--	Lysine	Cl	H	H	H
Ochratoxin A, alanine analogue	--	Alanine	Cl	H	H	H
Ochratoxin A, leucine analogue	--	Leucine	Cl	H	H	H
*d*-Ochratoxin A	*d*-OTA	d-Phenylalanyl	Cl	H	H	H
Ochratoxin A, ethylamide	OE-OTA	Phenylalanyl, ethylamide	Cl	H	H	H
*O*-methylated ochratoxin A	OM-OTA	Phenylalanyl, OCH_3_ at C-8	Cl	H	H	H
Methyl ester of ochratoxin α	M-OTα	Methoxy	Cl	H	H	H
Ochratoxin A, decarboxylated	DC-OTA	Phenylethylamine	Cl	H	H	H
***Other related compounds***						
Ochracin (=mellein)	--	H at C-7	H	H	H	H

The table contains only those forms that have been detected or synthesised and described until present. Summarised from [39,51,52,53,54,55,56,57].

### 2.2. Contamination Levels and Human Exposure

Ochratoxins occur ubiquitously and, at least for OTA, contamination levels in food and feed are monitored in many countries. OTB contamination levels of food and feed were assumed to be generally low [58], but based on the data on production described above, it can also be assumed that the contamination levels may sometimes be almost as high as those of OTA. Unfortunately, there is insufficient data on OTB contamination of food and feed, although when tested, OTB is often found together with OTA in both [59]. For example, a survey of Japanese commercial foods containing cereal, fruit, coffee or cacao showed a contamination with OTA at the low µg kg^−1^ level in more than one third of the samples [60]. Those with high OTA levels also contained OTB (up to 30% of the OTA concentration) [60]. Another survey of 681 samples from more than 50 different spice commodities resulted in positive detection of OTA and OTB in 21% and 10% of samples, respectively [61]. More recently, 57 samples of Sicilian red wines were found to be mostly weakly contaminated (<EU legal limit), ranging 0.02–0.73 μg L^−1^ and 0.04–0.66 μg L^−1^ for OTA and OTB, respectively [62]. OTA was found in 71.9% and OTB in 64.9% of samples tested [55]. Similar observations have been published for animal feed. For example, Hamilton and co-workers found a contamination ratio of about 90:8:2 for OTA, OTB and OTC, respectively, in poultry feed [13].

Humans are mainly exposed via food and beverages and the human dietary exposure has been estimated to be 15–60 ng OTA kg^−1^ b.w. per week for adult consumers in the EU [8,63]. Furthermore, many farm animals are fed with cereal-based diets exposing them to ochratoxins. Based on the available data on ochratoxin occurrence, it can be assumed that animals and humans are mainly exposed to OTA and to a much lesser extent to OTB and OTC. However, varying maximum exposure levels of all three forms as well as mixtures of different ratios may occur.

A recent human biomonitoring study of 33 mycotoxins in the Belgian population revealed that nine of these were detected via LC-MS/MS whereby deoxynivalenol, OTA, citrinin and their metabolites were the most frequently detected [64]. Citrinin and OTA were present in very low concentrations (pg mL^−1^ urine) and contaminated 59% and 35% of the samples from adults, respectively, with the rates in children being even higher [64]. The estimated daily intake of OTA showed that about 1% of the investigated population possibly exceeded the tolerable daily intake level [64].

### 2.3. Toxicokinetics of Ochratoxins

For the interpretation of *in vivo* data from toxicological reports an understanding of the kinetics of ochratoxins is crucial, including the absorption, distribution, metabolism, and excretion of the toxins [65]. The toxicokinetics of OTA and their toxicological implications for animals and humans have been extensively reviewed by various authors [7,39,53,66,67,68,69,70,71,72,73]. For example, the available kinetic data for rats indicate that strain, sex and age differences in OTA kinetics may account for the different sex and species sensitivities towards OTA [69]. Human data is rather limited, however, gender, season and geographic location seem to be most important factors [68].

Toxicokinetic studies employing ochratoxins other than OTA are rare [58,74,75,76]. For example, Mally and co-workers reported that OTB is more extensively metabolised and more rapidly eliminated than OTA in F344 rats due to the lack of specific retention of OTB in the kidneys, which may explain the different toxicological outcomes in animals compared to *in vitro* approaches [58].

**Figure 1:**
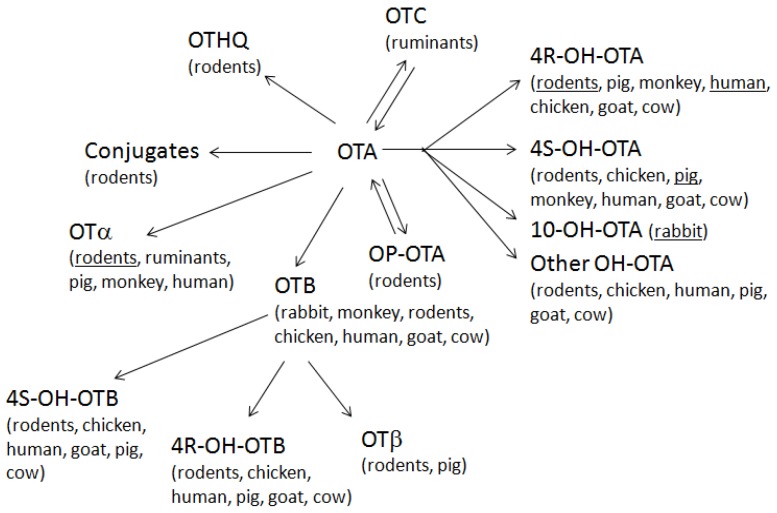
Overview of ochratoxin A (OTA) metabolites in animals and humans. Ochratoxins are differentially metabolised in various animals and humans depending on the metabolising enzymes present in liver, kidneys and/or gut. OTA, ochratoxin A; OTB, ochratoxin B; OTC, ochratoxin C; OTα, ochratoxin α; OTβ, ochratoxin β, OTHQ, OT hydroquinone; 4*R*-OH-OTB, 4*R*-hydroxyochratoxin B; OP-OTA, lactone-opened OTA; 10-OH-OTA, 10-hydroxyochratoxin A; 4*R*-OH-OTA, 4*R*-hydroxyochratoxin A; 4*S*-OH-OTA, 4*S*-hydroxyochratoxin A; 4*S*-OH-OTB, 4*S*-hydroxyochratoxin B. The respective metabolite is the main metabolite in the underlined taxa (for rodents, data of different authors is contradictory). Genera in brackets represent those where the metabolite has been detected, although it may be present in other animals or humans as well. Summarised from [52,53,92].

Based on the metabolism of animals and humans which includes hydrolysis, hydroxylation, lactone opening and conjugation [53], various ochratoxin metabolites can occur (Table 2, Figure 1). OTA can be enzymatically hydrolysed (e.g., by carboxypeptidase A, chymotrypsin) to the less toxic OTα by the bacterial microflora in the intestine as shown in ruminants and rodents [53,77,78]. Approximately 25% of ingested OTA is excreted as OTα in the urine of rats after reabsorption from the intestine [79,80]. OTα was also formed in OTA-exposed human bronchial epithelial cells *in vitro* [81].

Hydroxylated urinary metabolites of OTA include 4-hydroxy (4*R*- and 4*S*-) epimers, which are produced in liver [52,80,82] and kidney [80,83] by the action of various cytochromes P450 [80,82,84]. Exposed rats (*i.p.* or orally) excrete 4*R*-OH-OTA in their urine, together with the parent compound and OTα [85]. These 4-hydroxy metabolites are also produced by human, pig, goat, chicken and rat liver microsomes or human bronchial epithelial cells *in vitro* [52,80,81,82]. Furthermore, the 10-hydroxy metabolite is produced by rabbit liver and kidney microsomes and in human bronchial epithelial cells in culture, however, it has not yet been detected *in vivo* [80,81,84,86,87,88]. A recent study compared the *in vitro* metabolic profile of OTA in rat, chicken, pig, goat, cow and human liver microsomes [52]. This study resulted in the same six metabolites in all genera, *i.e.*, 4*S*-OH-OTA, 4*R*-OH-OTA, 7′-OH-OTA, 9′-OH-OTA, 5′-OH-OTA and OTB [52]. A semi-quantitative analysis showed that most of the OTA remained unchanged, confirming earlier reports that OTA is not primarily metabolised in the liver [52,53,79]. Furthermore, the metabolic capacity of human liver microsomes was higher compared to the other animals tested resulting in larger amounts of the metabolites [52]. Additional *in vivo* experiments in rats and chicken showed that OTA undergoes extensive metabolism after oral administration resulting in eight and six metabolites in rats and chicken, respectively [52]. These metabolites included 4*S*-OH-OTB and 4*R*-OH-OTB (in rats only) in addition to those found *in vitro* [52]. Again, parent OTA was the major compound in rat urine (64.0% ± 4.0%) and chicken excreta (56.0% ± 2.5%) [77]. The major metabolite was 4*R*-OH-OTA in rats (>20% *vs.* <10% in chickens) and 7′-OH-OTA in chickens (20% *vs.* 0.2% in rats) [52].

Glucuronidation of ochratoxins has been shown in rat liver microsomes [57] and *in vivo* in [^3^H]-OTA-exposed mice [89], where a large proportion of the biliary metabolites were ochratoxin glucuronides and sulphate conjugates [89]. Such conjugates were also detected in liver and intestinal tissue, although to a smaller percentage [89]. Also in pigs, glucuronide conjugates were detected in the bile [90]. In contrast, in *in vitro* experiments using primary rat and human hepatocytes, no ochratoxin glucuronide and sulphate conjugates were observed [91]. Pentose and hexose conjugates of OTA were detected in primary rat and human hepatocytes [91] and in the urine of rats [92]. Glutathione (GSH) conjugation is also an important mechanism in xenobiotic biotransformation and is generally a detoxification step, although it can result in bioactivation, in particular by formation of a thiyl radical, which favours the formation of peroxyl radicals. For example in *E. coli*, a cysteinyl-OTA conjugate has been detected [93]. Stable GSH-conjugates such as quinone-derived GSH-conjugates can be formed in the liver and can be biotransformed into cysteinyl conjugates in the kidney, which are then *N*-acetylated and excreted [94]. Most likely, GSH-conjugates are produced to a very low extent (1%) *in vivo* and, therefore, their detection is difficult and rarely reported [94,95].

Lactone-opened OTA (OP-OTA) was detected as a metabolite in the bile in OTA-exposed (*i.v.*) rats, but not in the blood or the urine [86], whereas other researchers found it in bile and urine of OTA-exposed (*i.v.*) rats [96]. The mechanism of OP-OTA synthesis as well as its potential role in bioactivation of OTA still needs to be elucidated [39].

In all investigations, parent OTA was always the main compound detected followed by species-specific metabolites.

### 2.4. Detection Methods for Ochratoxins

Knowledge about the different ochratoxin group members begins with the ability to detect and characterise them. OTA was detected first in 1965 [1] and, with improving detection methods, more and more ochratoxin family members are being described with reports of newly detected metabolites as recently as this year [52].

The analytical methods typically used have been reviewed by various authors in recent years [69,97,98,99]. These methods are able to detect and distinguish between the various ochratoxin family members and metabolites, but are technically and financially demanding. In contrast, immunological methods are typically less expensive but can detect only one specific ochratoxin or cannot distinguish between different ochratoxins as comprehensively reviewed by Meulenberg [100]. Most of these assays have been developed for the detection of OTA, however, at least one highly specific monoclonal antibody against OTB has also been successfully produced [101,102,103,104,105]. Although official monitoring programmes preferentially use analytical methods such as HPLC and LC-MS, immunological methods can be equally sensitive and reproducible [100].

## 3. Differential Toxicity of Ochratoxin Group Members

The toxicological profile of OTA has been investigated in numerous studies and also extensively reviewed [6,7,54,63,69,70,71,72,73,106,107,108,109,110,111,112,113,114,115,116,117,118,119,120,121,122,123,124,125,126,127,128,129,130,131,132,133,134,135,136,137,138]. In summary, these studies showed that OTA is nephrotoxic, hepatotoxic, neurotoxic, teratogenic and immunotoxic in various animals and *in vitro*, with renal toxicity and carcinogenesis being the key adverse effects. Many studies of OTA from the last decade support a non-DNA-reactive genotoxic mechanism, which involves different epigenetic mechanisms mainly related to oxidative stress, compensatory cell proliferation and disruption of cell signaling and division, and these publications have been thoroughly reviewed by various authors [6,63,70,113,119,139,140]. However, a direct genotoxic mechanism involving OTA bioactivation and DNA adduct formation is also still discussed [115,120] and this mechanism is also consistent with some *in vivo* gene expressionresults [113]. In summary, the mode(s) of action of OTA, in particular for renal carcinogenesis, are still under debate.

Various public health agencies and (inter)national authorities including the International Agency for Research on Cancer (IARC) [141], the European Food Safety Authority (EFSA) [8,142], the Joint FAO/WHO Expert Committee on Food Additives (JECFA) [128,143,144,145,146], the European Commission [5,147], the U.S. Department of Health and Human Services (National Toxicology Program, NTP) [148,149,150], and Health Canada [151,152] have evaluated the available data. Based on these evaluations, certain maximum limits for OTA in food have been set or proposed by the EU and Canada, ranging from 10 µg OTA kg^−1^ for dried fruits or instant coffee down to 0.5 µg OTA kg^−1^ for baby foods [6,147,153]. Egypt, Russia, China and Bosnia and Herzegovina have also established limits similar to that of European regulations [154,155]. OTA limits described in the *Codex Alimentarius* of the FAO/WHO have been adopted for example in the Arab States of the Gulf, Nigeria, Kenya and India. However, to date, no limits for OTA in foodstuffs have been set in many countries including the USA, Australia, New Zealand, Japan, Mexico or South Africa. Furthermore, there are no regulations for other ochratoxin group members in food and feed worldwide.

### 3.1. Comparative Toxicity of Ochratoxin Group Members

Comparative studies on more than two ochratoxins are very rare and no recent data are available. For example, Xiao *et al.* investigated toxicity using three different models [86]. In HeLa S3 cells, a cytotoxicity ranking (LC_50_ (µM)) of OTA (5) > OTC (9) > OTB (54) > *d*-OA (163) > OTα (560) > OM-OTA (830) > DC-OTA (7600) > OE-OTA (10,100) was found [86]. Similarly, the minimum toxic dose (nmol disc^−1^) was determined in an antibacterial assay using *Brevibacillus brevis*: OE-OTA (1.1) > OTC (2.0) > *d*-OTA (5.5) > OTA (8.7) > OTB (54) > M-OTα (90) > OTα (390) [86]. Furthermore, the *in vivo* toxicity was tested in mice (*i.p.*, 72 h) [86]. Here, no toxicity ranking could be established based on the chosen doses. At doses of 500 mg kg^−1^ b.w. all animals died within 72 h of dosing with OE-OTA, DC-OTA, OM-OTA or M-OTα, whereas there was no lethality at 200 mg kg^−1^ b.w. [86]. In comparison, OTA showed 30% and 90% lethality at 20 and 50 mg kg^−1^ b.w., respectively, and *d*-OTA showed no lethality at 50 and 200 mg kg^−1^ b.w. [86]. Further structure–activity analysis resulted in the conclusion that the toxicity of OTA is associated with its isocoumarin moiety and most likely with the lactone carbonyl group, but not with the phenyl hydroxyl group nor the iron-chelating properties [86]. In another study, Müller *et al.* compared the effects of different ochratoxin group members on extracellular radical formation in porcine blood monocytes and granulocytes and presented the following ranking (IC_50_ in ng mL^−1^ shown in parenthesis): OTA (89.1) > OTC (154.2) > OTA methylester (285.5) > OTB (>1000) = OTα (>1000) [156].

In summary, based on the limited data from the above studies, no clear general toxicity ranking can be drawn; however, OTA seems to be overall the most toxic, followed by OTC, OTB and OTα.

### 3.2. Ochratoxin B (OTB)

Most of the published data on ochratoxins other than OTA describes OTB toxicity. *In vivo*, OTB appears to be much less toxic than OTA as tested in rats, fish and poultry [58,157,158]. The first comparative study was performed in 1-day-old chicks resulting in oral LD_50_ values of approximately 120 µg OTA (about 3.5 mg kg^−1^) and 1890 µg OTB (54 mg kg^−1^) [157]. Another early *in vivo* study in rainbow trout resulted in an LD_50_ (*i.p.*) of 5.53 mg OTA kg^−1^ b.w., whereas no deaths were observed with doses up to 66.7 mg OTB kg^−1^ b.w. [158]. Mally and co-workers showed in F344 rats that oral administration of OTB did not induce pronounced adverse effects (minor renal histopathology, no changes in clinical markers for renal damage) [58] whereas the same treatment with OTA resulted in necrosis and degeneration of the renal tubular epithelium [159] and altered renal function [58]. The same authors also provided an explanation for the observed lower *in vivo* renal OTB toxicity compared to OTA: this is due to an absence of specific retention in the rat kidney which leads to its differential toxicokinetics *in vivo*, including its more extensive metabolisation combined with a more rapid excretion than OTA [58,74]. In contrast, whole-body autoradiography revealed no difference in OTA and OTB distribution in rats [74,75]. Additional *in vivo* data was provided by Chattopadhyay and colleagues in a recent hematotoxicity study in Sprague-Dawley rats (250–320 g) [160]. All chosen hematological parameters were significantly changed after *i.p.* injection of 0.5 μg OTB when compared to vehicle-treated animals [160].

Much more data is available from *in vitro* studies, where the nephrotoxic, hepatotoxic and the immunotoxic effects have been investigated. Early studies on *in vitro* toxicity reported OTB to be less toxic than OTA [161,162]. For example, serum-free *in vitro* cytotoxicity testing in embryonic chick meningeal fibroblasts resulted in significantly higher toxicity (nearly 19-fold) of OTA compared to OTB after eight days of exposure as measured using the MTT or NR assay [162]. Cytotoxicity was also investigated in continuous kidney cell lines (LLC-PK1 and OK) with four cytotoxicity assay endpoints resulting in OTA being more toxic than OTB [161]. The acute toxicity of OTA and OTB was compared in HeLa cells, resulting in LC_50_ values of 54 µM and 5 µM for OTB and OTA, respectively [86]. More recent investigations have resulted in a more detailed picture, with some endpoints or cell models resulting in a lower or at least different toxicity of OTB compared to OTA [163] while others have suggested an equal toxicity [58]. For example, in the human-derived liver cell line HepG2, OTA but not OTB caused induction of micronuclei and DNA migration, whereas OTB inhibited cell division at concentrations even lower than OTA [163].

Some studies directly compared OTA and OTB toxicity. An exposure to acutely toxic concentrations (µM range) of OTA and OTB typically resulted in a lower toxicity of OTB compared to OTA [58,161], which seemed to support the general belief that OTB is less toxic than OTA. However, this difference is typically only apparent after rather short exposure times, with differences becoming less significant the longer the exposure time. The degree of difference is also dependent on the cell type used. More detailed investigations of the acute effects (24–96 h) in different renal cells of human, porcine and rat origin resulted in a more complicated picture [164,165,166,167] (Table 3).

**Table 3:** Comparative cytotoxicity of OTA and OTB.

Cell Type (Gender)	96 h EC_50_			24–96 h EC_50_			48 h EC_50_
	NR	MTT	CN	NR	MTT	CN	CV
HKC (f)	OTA ↑	OTA ↑	OTA ↑	n.d.	n.d.	n.d.	OTA ↑
HKC (m)	Equal	Equal	Equal	Equal	Equal	Equal	OTA ↑
PKC (f)	Equal	Equal	Equal	n.d.	n.d.	n.d.	Equal
PKC (m)	Equal	Equal	Equal	Equal	Equal	Equal	Equal
RPTC (f)	OTA ↑	OTA ↑	n.d.	n.d.	n.d.	n.d.	n.d.
RPTC (m)	OTA ↑	OTA ↑	n.d.	n.d.	n.d.	n.d.	n.d.
LLC-PK1	OTA ↑	OTA ↑	Equal	OTA ↑	OTA ↑	Equal	OTA ↑
NRK-52E	n.d.	n.d.	n.d.	n.d.	n.d.	n.d.	OTA ↑
IHKE	n.d.	n.d.	n.d.	Equal	Equal	Equal	n.d.

HKC, primary human kidney cells; PKC, primary porcine kidney cells; RPTC, primary rat kidney cells; LLC-PK1, porcine renal cell line; NRK-52E, rat renal cell line; IHKE, human renal cell line; f, female; m, male; NR, neutral red uptake; MTT, MTT reduction assay; CN, cell number (direct counting); CV, cell number (crystal violet staining); OTA ↑, higher cytotoxicity of OTA compared to OTB; Equal, equal cytotoxicity of OTA and OTB; n.d., not determined; summarised from [166,167,168].

**Figure 2:**
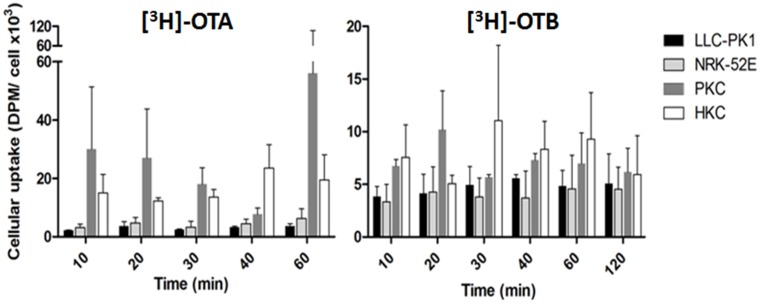
Comparison of cellular uptake of [^3^H]OTA and [^3^H]OTB. PKC, primary porcine kidney cells (male); HKC, primary human kidney cells (male). Columns represent means from at least three independent replicates ± SEM; modified from O’Brien *et al.* with permission [169].

Equal toxicity of both ochratoxins in exposed LLC-PK1 cells was corroborated by LC-MS/MS measurement of intracellular levels of ochratoxins showing no difference in intracellular concentrations of OTA and OTB, indicating that, provided equal amounts of toxin are taken up by the cells, OTA and OTB may be equally cytotoxic [58]. These authors therefore confirmed the earlier hypothesis, that the differential toxicities observed in some cell types are likely to be due to differences in uptake and cellular protein binding [58], as studied in various renal models. For example, cellular uptake of [^3^H]OTB was shown to be consistently lower than [^3^H]OTA uptake [169] (Figure 2). Furthermore, primary renal cells accumulated more toxins than continuous cell lines with the difference being more prominent with OTA [168]. Binding experiments showed that OTA has a binding affinity to a range of renal proteins from humans, pigs, mice and rats with species-specific binding patterns [169,170,171,172] (Table 4). Specific [^3^H]OTB binding was observed to be consistently lower than that of [^3^H]OTA, however, the same binding rank-order was determined for both toxins, *i.e.*, human > rat > pig > mouse [169,172]. Furthermore, the specific binding was at least 2.5 times higher with [^3^H]OTB, when compared with [^3^H]OTA [169,172].

**Table 4:** Comparison of [^3^H]OTA and [^3^H]OTB protein binding characteristics.

Species (Gender)	[^3^H]OTA			[^3^H]OTB			*p*
	SB (pmol mg^−1^ Protein)	SB (%)	*n*	SB (pmol mg^−1^ Protein)	SB (%)	*n*	
Human (m)	21.4 ± 4.5	20.5 ± 4.1	6	1.25 ± 0.15	52.9 ± 10.7	3	0.0002
Human (f)	23.1 ± 4.7	22.0 ± 4.3	4	3.79 ± 2.04	73.6 ± 4.0	3	<0.0001
Rat (m)	10.2 ± 3.7	10.1 ± 3.4	14	0.44 ± 0.42	31.2 ± 15.0	8	<0.0001
Rat (f)	14.2 ± 6.3	11.5 ± 4.7	10	0.73 ± 0.39	43.3 ± 11.4	6	<0.0001
Pig (m)	3.4 ± 1.0	3.3 ± 0.9	8	0.19 ± 011	27.2 ± 13.0	5	0.0002
Pig (f)	2.1 ± 0.7	2.0 ± 0.6	8	<LOD	<LOD	2	n.c.
Mouse (m)	0.9 ± 02	0.9 ± 0.2	6	0.16	14.3	1	n.c.
Mouse (f)	1.5 ± 0.6	1.5 ± 0.5	9	<LOD	<LOD	2	n.c.

Means ± SD; SB, specific binding; n, number of replicates; LOD, limit of detection; *p* values are shown from two-tailed *t*-tests and refer to the comparison of SB(%); n.c., not computable; modified from O’Brien *et al.* with permission [169].

The binding data obtained *ex vivo* correlated well with the *in vivo* toxicokinetic profiles of OTA which were studied for example in fish, quail, mouse, rat and monkey [76]. The elimination half-life varied from 0.68 h after oral administration to fish, up to 840 h after intravenous administration to monkey [76]. A comparative study in rats showed that the biological half-life (t_1/2_) as well as the maximal serum concentration was different with OTA and OTB [70]. OTA showed a t_1/2_ of 170 h (*i.v.*) or 120 h (*p.o.*), whereas that of OTB was much shorter, *i.e*., 12 h (*i.v.*) or 18 h (*p.o.*) [76]. The maximal serum concentrations were 2100 ng mL^−1^ (*i.v.*) or 390 ng mL^−1^ (*p.o.*) and 760 ng mL^−1^ (*i.v.*) or 120 ng mL^−1^ (*p.o.*) for OTA and OTB, respectively. Furthermore, the binding parameters were determined by equilibrium dialysis resulting in unbound fractions of ochratoxins in rat (0.02% OTA *vs.* 0.9% OTB), monkey (0.08% OTA *vs.* 2.7% OTB), and humans (0.02% OTA *vs.* 2% OTB) [76]. Plasma protein binding of OTA was studied in various animals after the oral or intravenous administration of 50 ng g^−1^ b.w. resulting in a free fraction of toxin in plasma of <0.2% in all subjects investigated except fish where the equivalent figure was 22% [76].

Dietrich and colleagues performed further testing with respect to the reversal of toxic effects. After 48 h of exposure (10 µM) with subsequent replacement of the medium, they demonstrated recovery from the cytotoxic effect for OTA in some cell models, *i.e.*, in LLC-PK1, NRK-52E and NRK-49F, but not in human and porcine primary kidney cells [166]. In contrast, only the fibroblast cells (NRK-49F) were able to recover from the toxic effects of OTB [168]. These observations further support the conclusion of differential cytotoxicity of OTA and OTB in different cell models.

Comparative studies were also undertaken with respect to chronic toxicity. For this, an *in vitro* model of sub-chronic repeated exposure to OTA and OTB was employed to generate ochratoxin-derived subpopulations of human and porcine proximal tubular cells (HKC, IHKE, PKC, LLC-PK1) [167,173,174,175]. Comparison of growth characteristics showed marked changes in almost all cell types, however, a comparison between OTA and OTB showed no difference between the different cell subpopulations [167]. Acute challenge experiments with these cell subpopulations resulted in generally decreased sensitivity towards OTA- and OTB-mediated cytotoxicity [167].

The immunotoxic effects of OTB were studied in isolated human neutrophils where all parameters studied were decreased at µg mL^−1^ concentrations, indicating a weakened immune defence [176,177]. In porcine monocytes, extracellular radical formation was inhibited by OTA almost completely at 1 µg mL^−1^, whereas the inhibition by OTB was lower [156]. OTA impaired the IL-2 production and IL-2 receptor expression of activated T lymphocytes, which was prevented when the cells are pre-incubated with OTB prior to OTA exposure [178].

Other comparative toxicity investigations included the examination of mutagenicity and genotoxicity. For example, OTA induced SOS-DNA repair without metabolic activation at concentrations of 0.1–4 mM in *E. coli*, whereas OTB up to 4 mM was inactive [93,179]. The same was shown for induction of micronuclei and DNA migration assays, whereas both toxins failed to induce mutations in *Salmonella*/microsome assays [163].

Differential toxicity has also been shown for OTA and OTB in developmental toxicity models. OTB and OTA both caused craniofacial malformations in the FETAX (Frog Embryo Teratogenicity Assay *Xenopus*) with the effects of OTA being more severe than those of OTB [180]. OTA also caused reduced embryo growth, whereas OTB did not [180]. The authors suggested that this differential embryotoxicity is at least in part due to the higher levels of OTA being accumulated within the embryos as shown by uptake experiments using [^3^H]OTA and [^3^H]OTB [180]. Investigations by other authors revealed that OTA and OTB also inhibited differentiation and protein synthesis in prechondrogenic mesenchymal cells from the limb buds of 4-day chick embryos with IC_50_ values of 1.9 µM and 6.2 µM for OTA and OTB, respectively [181]. Furthermore, OTB had no effect on the aminoacylation of phenylalanine tRNA catalysed by mice liver phenylalanyl-tRNA synthetase, whereas OTA showed a concentration-dependent effect [182]. Furthermore, OTB showed no antagonistic effect on the *in vivo* protein synthesis inhibition caused by OTA in hepatoma tissue culture cells [182].

In summary, today’s knowledge on comparative toxicity of OTA and OTB must differentiate between *in vivo* and *in vitro* effects. OTB is clearly less toxic *in vivo* compared to OTA as shown in various models. It has been shown that OTB is more readily eliminated and had a lower affinity for plasma proteins, which partly may explain its lower toxicity. In contrast, both toxins can be similarly acutely cytotoxic *in vitro* provided that similar amounts are taken up and are intracellularly bound, although several results may suggest a different molecular mechanism of chronic toxicity compared to OTA. Furthermore it can be assumed that the small structural difference (chlorine in OTA *vs.* hydrogen in OTB) although not responsible for the toxicity, may be crucial for the differential uptake and binding in cells.

### 3.3. Ochratoxin C (OTC) and Other Ethyl or Methyl Esters of Ochratoxins

In an early study, oral LD_50_ values were reported for OTC (216 mg animal^−1^) and OTA (166 mg animal^−1^) in day-old chicks [183]. Later, the acute cytotoxicities of OTA and OTC were compared in HeLa S3 cells, resulting in comparable LC_50_ values of 9 µM and 5 µM for OTC and OTA, respectively [86]. Furthermore, an antibacterial assay with *B. brevis* resulted in minimum toxic doses (MTDs, nmol disc^−1^) of 8.7 ± 1.7 and 2.0 ± 0.5 for OTA and OTC, respectively [86]. OTC was also identified as comparably cytotoxic to OTA in human embryonic (HEK293) and renal carcinoma (A498) cell lines [184]. Based on the above results, OTC is considered to be similarly toxic to OTA.

Further data have shown that OTC and OTA exposure leads to various immunomodulatory effects (e.g., metabolic activity, mitogen-induced proliferation, ROS formation, production of the cytokines IL-6 and TNF-α, phagocytic behaviour, nitrogen oxide synthesis and cell surface markers) in porcine mononuclear cells [185] and in the human monocyte/macrophage line THP-1 [186] at low concentrations (10–1000 ng mL^−1^). Similar concentrations of OTA and OTC suppressed significantly the formation of free oxygen radicals by porcine monocytes and granulocytes [156]. Long-term exposure of THP-1 cells to OTC and OTA (15 days, 1 ng mL^−1^) resulted in increased mitochondrial activity and production of IL-6, disturbed cell membrane integrity and inhibited cell proliferation and TNF-α and IL-8 production [187].

An early *in vivo* study in young rainbow trout with ethyl esters of OTA and OTB showed that these compounds were lethal to trout when administered *i.p.* over 10 days with LD_50_ values of 3.0 and 13.0 mg kg^−1^ b.w. for OTA and OTB ethyl esters, respectively [158]. OTα ethyl ester was non-toxic at a level of 3.9 mg kg^−1^ b.w., which is equivalent to 13.72 µmol kg^−1^ (=LD_50_ of OTA) [158]. In comparison to OTA, the methyl ester of OTA was less toxic than OTA in day-old chicks [183]. Also, OTB methyl and ethyl esters were found to be non-lethal to orally exposed day-old ducklings [188].

In summary, OTC seems to be similarly acutely toxic *in vivo* and *in vitro* compared to OTA, however, whether the mode of action of OTC and OTA is the same remains to be elucidated. Other ochratoxin ethyl or methyl esters show lower toxicity compared to OTA.

### 3.4. Ochratoxin α (OTα) and Ochratoxin β (OTβ)

Unfortunately, there are not many published studies including data on OTα and OTβ and most of the available data considers OTα only. For OTβ, only its occurrence as metabolite has been investigated. To our knowledge, no toxicity data is available. However, based on its structure, it may be assumed that OTβ shows similar behaviour to OTα.

Creppy and co-workers investigated the immunotoxic effect of OTα in BALB/c mice using an immunosuppression assay after exposure to a single dose of OTα of 1 µg kg^−1^ *i.p.* [85]. Here, OTA caused an approximately 90% reduction of the IgM and IgG response, whereas OTα showed no effect [85].

The same researchers reported that the growth rate of Morris hepatoma cells (HTC cells) was massively decreased with OTA, whereas OTα had no effect on proliferation [189]. The acute toxicity was also compared in HeLa cells, resulting in EC_50_ values of 560 µM and 5 µM for OTα and OTA, respectively [86]. A recent report compared the acute cytotoxicity (24 h) in IHKE cells and calculated an EC_50_ of 0.5 µM for OTA, whereas OTα was non-toxic up to 50 µM [190].

The neurotoxic effects were also investigated with OTA showing pronounced inhibition of various parameters (dehydrogenase activity, lysosomal activity, total cell culture protein, neurofilament 68 kD content) in cultures of embryonic brains of Tetra SL chick stage 29 at low nM concentrations and 8 days of exposure, whereas OTα showed no effect up to 15 µM [55]. In addition, ochracin (also termed mellein) was tested, resulting in weak cytotoxic effects at high concentrations (µM-mM) [55].

Others investigated genotoxic effects, resulting in induction of SOS-DNA repair in the *E. coli* PQ37 strain in the absence of an exogenous metabolic activation system at concentrations of 0.1–4 mM OTA, whereas OTα was inactive [179]. OTA induced also sister chromatid exchanges in cultured primary porcine urinary bladder epithelial cells (PUBEC) after 5 h of exposure at pM to nM concentrations without apparent cytotoxicity [191]. The same effect was observed with OTα, although higher concentrations were necessary (up to 10 µM) [191].

OTα is much less toxic (approximately 100 fold) than OTA as shown in various investigations [85,86]. Apparently, the isocoumarin moiety alone is not effective but must be bound to phenylalanine to exert toxic effects.

### 3.5. Hydroxyochratoxins

Also, for hydroxyochratoxins, only a very limited amount of data exists. Some data exist for 4*R*-OH-OTA: this was shown to be equally cytotoxic compared to OTA in Morris hepatoma cells (HTC cells) and this toxicity could be prevented by phenylalanine [189]. Furthermore, both OTA and 4*R*-OH-OTA inhibit protein synthesis, which could be prevented by phenylalanine and could be attributed to their binding on the phenylalanine sites of phenylalanyl-tRNA synthetase [189]. The same group also investigated the immunotoxic effects in BALB/c mice using an immunosuppression assay, resulting in similar toxic effects of 4*R*-OH-OTA and OTA on the IgM and IgG response [85]. 4-OH OTA resulted in no mortality after six days when injected *i.p.* into male Wistar rats at a dose of 40 mg kg^−1^ b.w. [192]. 10-OH OTA was found to be non-genotoxic (DNA adduct formation) in bronchial epithelial cells (WI 126 VA) [81,88]. Finally, no toxicity data are available for 4*R*-hydroxyochratoxin B (4*R*-OH-OTB) and 4*S*-hydroxyochratoxin B (4*S*-OH-OTB) to date. Other hydroxyochratoxins (5′-OH-OTA, 7′-OH-OTA, 9′-OH-OTA), which differ from OTA in the hydroxylation at specific sites of the phenylalanyl group, were detected as metabolites *in vitro* and *in vivo* just very recently [52] and no toxicity data are available to present.

Based on some of the results above, it may be concluded that hydroxylation of ochratoxins (in particular 4*R*-OH-OTA) does not obviously affect their toxicity.

### 3.6. Ochratoxin Quinone (OTQ)/Hydroquinone (OTHQ)

It has been shown that OTA undergoes an oxidative dechlorination process to generate a quinone (OTQ)/hydroquinone (OTHQ) redox couple like other chlorinated phenols, which may play a role in OTA-mediated toxicity [56,193]. Indeed, OTHQ generated DNA adducts in the absence of metabolic activation in human bronchial epithelial (WI26) and human kidney (HK2) cells. These results were obtained using the [^32^P]-postlabeling technique and the necessary confirmation by mass spectrometry is still lacking [56]. However, other researchers did not find OTAQ/OTAHQ metabolites in liver and kidney microsomes from rat or mouse [87,194].

In summary, data on OTAQ/OTAHQ metabolites are still too limited to provide meaningful conclusions.

### 3.7. Lactone-Opened OTA (OP-OTA)

The *in vivo* toxicity of OP-OTA was tested in rats and mice. OP-OTA was more toxic than OTA in rats when administered *i.v.* (3/4 rats died within 6 h *vs.* 2/4 with OTA at 500 µg animal^−1^ (200 ± 15 g b.w.) [86]. Interestingly, no lethality was observed after *i.p.* injection [86]. The same effect was observed in mice (6 weeks old, *n* = 10), where no lethality after 72 h at *i.p.* doses up to 500 mg kg^−1^ b.w. occurred, whereas in the same experiments OTA resulted in 90% dead animals after injections of 50 mg kg^−1^ b.w. [86]. These authors suggested that the more polar OP-OTA is, the less readily it is taken up in the rodents when given *i.p.* compared to OTA [86]. Whether other factors may be involved such as enhanced metabolism (first pass effect) or elimination was not investigated.

In addition, no toxicity was detected in *Brevibacillus brevis* at a concentration of 350 nmol/disc, whereas the minimum toxic dose for OTA was 8.7 ± 1.7 nmol disc^−1^ [86].

Overall, the available data suggests that the toxicity of ochratoxins may be related to the lactone carbonyl group.

### 3.8. Conjugated Ochratoxin Forms

To date, no toxicity data has been reported for glucuronidated ochratoxins or pentose and hexose conjugates. Only in bacteria (*E. coli* PQ37), the formation of a cytotoxic thiol-containing derivative was suggested [93]. Conjugation is typically a detoxification step, however, bioactivation might be possible.

### 3.9. Synthetic OTA Analogues

Investigations with synthetic analogues of OTA mostly included substitutions of phenylalanine with other amino acids. For example, in an early study, the alanine and leucine analogues of OTA were *i.p.* administered to trout and caused no death at dose levels of 4.49 and 5.06 mg kg^−1^ b.w., respectively [158]. These doses were equivalent to 13.72 µmol OTA kg^−1^ b.w. (=LD_50_ of OTA) [158]. Later, Creppy *et al.* synthesised eight different analogues of OTA [195]. Their effects were investigated in comparison to OTA, OTB and OTα on yeast tRNA amino acylation and on growth and protein synthesis of HTC cells [195]. All analogues showed inhibitory effects in the three test systems, although to a lesser degree than OTA, whereas OTB and OTα were ineffective [195].

Based on these results, it can again be concluded that phenylalanine is important for the toxicity of OTA, as the toxicity was always reduced if phenylalanine was substituted by another amino acid.

### 3.10. Structure–Activity Relationship

In summary, current knowledge about the toxicity of ochratoxins suggests that the hydroxyl, carboxyl, chlorine, and lactone groups of ochratoxins substantially affect their toxicities in various models. Furthermore, the biological reactivity of ochratoxins seems to be enhanced by the presence of the phenylalanine moiety, although this group is not essential for toxicity.

## 4. Toxicity of Mixtures of Mycotoxins

In nature, co-occurrence of mycotoxins is often observed. One mould species may produce many different mycotoxins, and the same mycotoxin may be produced by several species [110,196,197,198,199,200]. Considering this coincident production, it is very likely that humans and animals are always exposed to mixtures rather than to individual compounds. This is particularly true for OTA, OTB, citrinin, penicillic acid and occasionally for patulin as these are all produced by a number of *Penicillium* and *Aspergillus* species [110,200]. Furthermore, other mycotoxins such as fumonisins and aflatoxins have been frequently detected together with OTA [62,110,199]. Currently, little is known about mycotoxin interactions although combined exposure is clearly more relevant to real-life situations [2,3,110,114,200]. Further challenges may occur worldwide due to climate change as this may influence the occurrence of mycotoxins due to geographic shifts in the distribution of major cereal cropping systems and potentially favourable climatic conditions for toxin producing organisms [201,202].

Data on combination toxicity is still rather limited and often contradictory, however, the available data on OTA toxicity in combination with other mycotoxins was comprehensively reviewed in 2013 [110]. In summary, most of the studies addressing the combined effect of OTA with other mycotoxins showed additive or synergistic interactions when binary mixtures were tested and data on multiple mixtures is very rare [110]. Since the publication of that review, a few more reports have been published and these are summarised below [203,204,205,206,207].

Rumora *et al.* investigated the individual and combined toxicity of OTA and citrinin in porcine proximal tubular cells (PK15 cells) and showed that mitogen-activated protein kinases (MAPKs) were differentially activated [203]. Based on their results, the authors suggested that Ca^2+^ might be an important player in the observed *in vitro* toxicity [203]. These toxins were also tested by Anninou *et al.* on human hepatoma cells (Hep3B), in combination with sterigmatocystin, a precursor of aflatoxin biosynthesis, which resulted in additive or antagonistic genotoxic and cytotoxic effects at low concentrations (pM-nM) [208]. Föllmann and colleagues tested OTA in combination with citrinin and its main human metabolite dihydrocitrinone (DH-CIT) in Chinese hamster lung fibroblasts (V79 cells) using the neutral red uptake assay, the *in vitro* micronucleus assay and flow cytometry [205]. The main outcome of this study, *i.e.*, that mixtures of OTA and citrinin exerted mainly additive effects and that synergism was observed with high micromolar concentrations only [205], confirms some of the earlier results obtained in other cells and/or with other endpoints [209,210,211,212]. Similar results with OTA and citrinin were obtained by Gayathri *et al.* in human hepatocarcinoma cells (HepG2) [213].

Clarke and co-workers investigated OTA in combination with aflatoxin B_1_ (AFB_1_) and fumonisin B_1_ (FB_1_) in human intestinal cells (Caco-2), Madin Darby bovine kidney cells (MDBK) and murine macrophage cells (Raw 264.7) using the MTT and neutral red assays [207]. Binary combinations showed additive effects, whereas tertiary combinations of OTA, FB_1_ and AFB_1_ resulted in synergistic effects [207]. Further studies employed more sensitive high content analysis (HCA) resulting in synergistic interactions of binary mixtures of OTA and FB_1_ [204]. In contrast, recent *in vivo* investigations by Corcuera *et al.* of the combined toxicity of OTA and AFB_1_ in orally exposed male F344 rats resulted in an antagonistic genotoxic interaction as tested via the micronucleus and comet assays [214].

Li and co-workers investigated the liver and ovarian toxicity of OTA in combination with the mycotoxin zearalenone in human hepatoma cells (HepG2) and murine ovarian granular cells (KK-1) [206]. Exposure to equi-effective mixtures resulted in additive effects with respect to cell viability (WST-8 assay), whereas differential effects were observed with the endpoint of ROS generation [206]. Further research included also other targets such as intestinal cells or lung cells, for example, Cano-Sancho and co-workers reported an increased cytotoxicity of binary combinations of OTA and deoxynivalenol on Caco-2 cells [215]. Šegvić Klarić *et al.* investigated subtoxic spore extracts from typically co-occurring grain mill aeromycota in combination with OTA on human lung adenocarcinoma cells (A549) and observed significant DNA damage and cell death with additive interactions [216].

In summary, the combined effect of OTA with other mycotoxins showed mostly additive or synergistic interactions. Depending on the concentrations applied and the *in vitro* models employed, however, antagonistic interactions have also been detected. Data on multiple mixtures are still incomplete.

## 5. Summary and Final Remarks

The biosynthesis as well as biotransformation of ochratoxins has not been elucidated in detail so far. Several naturally occurring forms, metabolites and conjugates have been reported and characterised *in vitro* and/or *in vivo*, while the toxicity of other detected forms remains to be described. Based on the publication record on ochratoxin metabolism, it can be concluded that humans and animals are mainly exposed to the ingested parent compounds and to a much lesser extent to their (species-specific) metabolites.

The current toxicological database indicates the crucial importance of certain structural components such as the hydroxyl, carboxyl, chlorine, and lactone groups of ochratoxins with respect to cellular uptake and binding. The bioavailability seems to be further enhanced by the presence of the phenylalanine moiety, although this group is not essential for toxicity. The structural differences of the ochratoxin group members may explain most of the experimental outcomes observed in various models. Nevertheless, the contribution of metabolites to OTA toxicity is still rather unclear and further research is needed.

The underlying mechanisms of toxicity of the ochratoxin group members still need to be elucidated. Although recent advances mainly support a non-DNA-reactive, genotoxic mechanism for OTA, there is also evidence of other mechanisms involving OTA bioactivation and OTA-DNA adduct formation. Whether or not other ochratoxin group members show the same mode of action remains to be investigated. However, there are indications that at least OTB may have a different molecular mechanism of chronic toxicity compared to OTA.

Given the potential chronic human exposure to the naturally occurring forms, metabolites, conjugates and mixtures with other mycotoxins, a better understanding of the toxicokinetics and the mechanisms of toxicity of the ochratoxin group is necessary to provide adequate human risk assessments.

## Abbreviations

b.w., body weight; (B)EN, (Balkan) Endemic Nephropathy; GSH, glutathione; FETAX, Frog Embryo Teratogenicity Assay *Xenopus*; CAS, Chemical Abstracts Service; EC_50_, mean effective concentration; EU, European Union; FAO, Food and Agriculture Organization of the United Nations; IgG/M, immunoglobulin G/M; *i.p.*, intraperitoneal; *i.v.*, intravenous; LC_50_, median lethal concentration; LD_50_, median lethal dose; MPN, Mycotoxic Porcine Nephropathy; *p.o.*, *per os* (orally); MTT, 3-(4,5-dimethylthiazol-2-yl)-2,5-diphenyltetrazolium bromide; WHO, World Health Organization.
